# Intensive insulin therapy increases glutathione synthesis rate in surgical ICU patients with stress hyperglycemia

**DOI:** 10.1371/journal.pone.0190291

**Published:** 2018-01-04

**Authors:** Gianni Biolo, Benedetta Massolino, Filippo Giorgio Di Girolamo, Nicola Fiotti, Filippo Mearelli, Sara Mazzucco, Carlos Bertuzzi, Renzo Lazzarini, Alfonso Colombatti, Marcello De Cicco

**Affiliations:** 1 Department of Medical, Surgical and Health Sciences, University of Trieste, Clinica Medica, ASUITS, Trieste, Italy; 2 Intensive Care Unit, Centro di Riferimento Oncologico, Aviano, Italy; 3 Hospital Pharmacy, Centro di Riferimento Oncologico, Aviano, Italy; 4 Experimental Oncology 2, Centro di Riferimento Oncologico, Aviano, Italy; National Yang-Ming University, TAIWAN

## Abstract

**Objective:**

The glutathione system plays an essential role in antioxidant defense after surgery. We assessed the effects of intensive insulin treatment (IIT) on glutathione synthesis rate and redox balance in cancer patients, who had developed stress hyperglycemia after major surgery.

**Methods:**

We evaluated 10 non-diabetic cancer patients the day after radical abdominal surgery combined with intra-operative radiation therapy. In each patient, a 24-hr period of IIT, aimed at tight euglycemic control, was preceded, or followed, by a 24-hr period of conventional insulin treatment (CIT) (control regimen). Insulin was administered for 24 hours, during total parenteral nutrition, at a dosage to maintain a moderate hyperglycemia in CIT, and normoglycemic blood glucose levels in IIT (9.3±0.5 vs 6.5±0.3 mmol/L respectively, P<0.001; coefficient of variation, 9.7±1.4 and 10.5±1.1%, P = 0.43). No hypoglycemia (i.e., blood glucose < 3.9 mmol/L) was observed in any of the patients. Insulin treatments were performed on the first and second day after surgery, in randomized order, according to a crossover experimental design. Plasma concentrations of thiobarbituric acid reactive substances (TBARS) and erythrocyte glutathione synthesis rates (EGSR), measured by primed-constant infusion of L-[^2^H_2_]cysteine, were assessed at the end of each 24-hr period of either IIT or CIT.

**Results:**

Compared to CIT, IIT was associated with higher EGSR (2.70±0.51 versus 1.18±0.29 mmol/L/day, p = 0.01) and lower (p = 0.04) plasma TBARS concentrations (2.2±0.2 versus 2.9±0.4 nmol/L).

**Conclusions:**

In patients developing stress hyperglycemia after major surgery, IIT, in absence of hypoglycemia, stimulates erythrocyte glutathione synthesis, while decreasing oxidative stress.

## Introduction

Stress hyperglycemia, associated with insulin resistance, is a common consequence of major surgery, both in patients with, or without, diabetes mellitus [1]. Van der Berghe et al. showed that an intensive insulin treatment (IIT), targeted at blood glucose level of 4.4–6.1 mmol/L, in intensive care unit (ICU) post-surgical patients, can improve their outcome [2]. Subsequent trials [3–7], however, showed no benefits, or even increased mortality, from such tight glucose control. [3–8]. Current guidelines recommend to apply, in both critically and non-critically ill ICU patients, with blood glucose levels >180 mg/dL (10.0 mmol/L), a conventional insulin treatment (CIT), aimed at a target glycaemia between 140–180 mg/dL (7.8–10.0 mmol/L) [9–11].

Other authors however, suggest that IIT efficacy, in improving outcomes, depends mostly on the patient’s clinical conditions, being more beneficial in surgical vs non-surgical ICU patients and in non-diabetic and non-critically ill hospitalized cases [1,12–15]. Improved outcome associated with IIT in surgical patients appears to be mainly the result of a reduced risk of hospital-acquired infections [1,12–16]. Continuous blood glucose–monitoring devices and computer-assisted closed loop systems for insulin delivery can prevent hypoglycemia and limit glucose level variability [17]. These methods have been recently applied to test the effects of a tight glucose control after hepatobiliary-pancreatic surgery [18]. Compared to CIT, IIT was associated with a 58% and 54% reduction respectively in surgical site infections and pancreatic fistula [18].

Oxidative stress can impair immunity and increase complications induced by glycemic variability, such as infections and organ dysfunction [19]. Insulin deficiency and hyperglycemia can directly increase free radical production and impair antioxidant defenses [20]. The tripeptide glutathione is the most important endogenous antioxidant in humans [20]; its synthesis starts with the formation of the dipeptide gamma-glutamyl-cysteine, regulated by the glutamyl-cysteinil ligase, followed by the addition of glycine, catalyzed by glutathione synthetase, [20]. The glutathione availability depends on glutathione peroxidase, which catalyzes the formation of glutathione disulfide (dimeric form), from monomeric glutathione, during hydroperoxide reduction, and glutathione reductase, that regenerates the monomeric active form. Stress hormones and cytokines can down-regulate glutathione synthesis, while insulin up-regulates gamma-glutamyl-cysteinil ligase [20–24]. In addition, cysteine, the key precursor amino acid, is the rate-limiting metabolite [20]. Glutathione availability has been shown to be decreased in surgical trauma and other acute illnesses, as well as in chronic diseases, including diabetes and cancer [20,21]. Glutathione depletion has been clearly associated with cell damage and apoptosis in vitro [22] and with increased mortality in clinical trials [20].

In this study, we aim to test the hypothesis that in patients with insulin resistance, after major surgery, IIT, compare to CIT, would enhance glutathione synthesis and/or availability and decrease systemic oxidative stress. To maximize insulin resistance, we have selected a population of well-nourished cancer patients, who had undergone abdominal radical surgery combined with intra-operative radiation therapy. We have designed a 2-day randomized, cross-over study to compare the effects of moderate hyperglycemia or euglycemia, induced by 24 hours CIT or IIT given in randomized order [25], on glutathione synthesis in erythrocytes -as determined by stable isotopic tracers [26],- and on plasma thiobarbituric acid reactive substance (TBARS) concentrations -as marker of lipid peroxydation [27],

## Methods

### Patients

We have enrolled 10 adult cancer patients (Table 1), with no other ongoing morbidities, from the “Centro di Riferimento Oncologico, Istituto Nazionale Tumori” (IRCCS, Aviano, Italy), admitted to ICU, after radical abdominal cancer surgery and intra-operative radiation therapy [25]. The experimental protocol was approved by the ethical committee of the IRCCS. Written informed consent was obtained from all patients before surgery. Protocol safety end-points included hypoglycemia (glycaemia ≤ 79 mg/dl) and/or hypokalemia (kalemia ≤ 3.0 mg/dl). Any patient reaching one of these end-points would have been excluded from the study.

**Table 1:** Patient characteristics.

Patient #	Gender (m/f)	Age (yrs)	BMI (kg/m^2^)	Diagnosis	Protocol^a^
1	m	53	21.6	colorectal cancer	2
2	m	50	29.1	colorectal cancer	2
3	m	52	23.4	colorectal cancer	1
4	m	70	26.5	colorectal cancer	1
5	f	54	27.7	colorectal cancer	2
6	m	73	25.9	retroperitoneal sarcoma	2
7	m	68	30.4	gastric cancer	1
8	m	69	21.1	gastric cancer	1
9	f	67	22.0	retroperitoneal sarcoma	1
10	f	36	23.3	endometrial cancer	2

^a^, Protocol 1 (n = 5) = Intensive Insulin Therapy (IIT) first; Conventional Insulin Therapy (CIT) second. Protocol 2 (n = 5) = CIT first; IIT second

**Fig 1:**
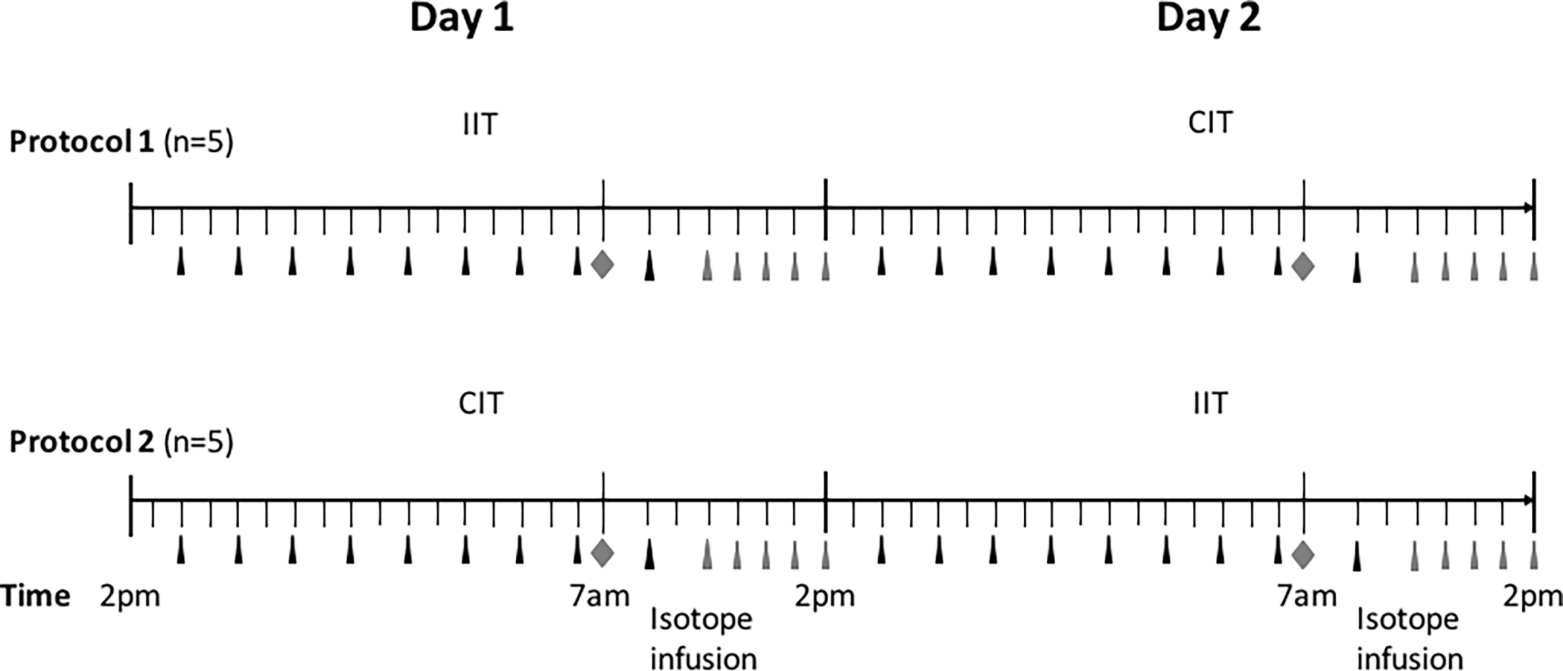
Experimental procedure. The experimental procedure started at 2 PM, the day of surgery, and continued for the next 48-h, inclusive of the 24-h of IIT and the 24-h of control CIT, administered according to *randomi*zed, cross-over protocols. Patients (n = 5) assigned to protocol 1 started with IIT, followed by CIT, while patients (n = 5) assigned to protocol 2 started with CIT followed by IIT. Throughout the 2-day study, glycaemia was determined every 2 hours (**▲**). The stable isotope infusion to determine erythrocyte glutathione kinetics and oxidative stress, was performed during the last 7 h of insulin treatment, either IIT or CIT (i.e. from 7 AM to 2 PM). Shortly before starting the infusion (◆) a blood sample was taken to assess background isotope enrichments and hematochemical and oxidative stress indices. Blood samples were taken at time 3,4,5,6,7 hour (▲) to assess the glutathione kinetics.

Experimental design: The full experimental design is reported in our previous paper [25]. Briefly, all patients received a standard continuous intravenous infusion of glucose (20% solution at 20 mL/kg/day), lipids (Intralipid 10% at 8 mL/kg/day) and mixed amino acids (Freamine III 8.5% at 14.4 mL/kg/day), for a total energy intake of 28 kcal/kg/day. The experimental procedure (Fig 1) started the day of surgery, at 2 PM, to precede over the next two 24 hour periods. Patients were assigned, according to a randomized crossover design, to either IIT (insulin infused if glycaemia > 6.1 mmol/L_,_ at dosages adjusted to maintain euglycaemia between 4.4–6.9 mmol/L) [2,3] or CIT, as control treatment (insulin given if glycaemia > 9.4 mmol/L at dosages adjusted to maintain a moderate hyperglycaemia, between 8.3–11.1 mmol/L). Five cases followed protocol 1 (i.e. IIT first, CIT second) and 5 cases protocol 2 (i.e. CIT first, IIT second). Such a randomization was performed to account for possible time-related changes in glutathione kinetics and oxidative stress, after surgery, and for the potential risks of interference between the two stable isotope infusions, administered with such a tight schedule [25]. Throughout the 2-day insulin infusion study, glycaemia was assessed every 2 hours. The stable isotope infusion to determine erythrocyte glutathione kinetics and oxidative stress, was performed during the last 7 h of insulin treatment (from 7 AM to 2 PM), in both IIT or CIT [25]. In order to assess clinical stability between the two isotope infusions, hemodynamic indices (systolic and diastolic arterial pressure, heart rate and hematocrit) and plasma creatinine concentrations, were determined before starting the infusion procedures.

### Isotope infusion procedure

Indwelling catheters, already placed for clinical purposes, in an internal jugular vein and in a radial artery, were used for isotope infusion and blood samplings. At 7 A.M., after background blood sampling, a primed-continuous infusion of 3.2±0.3 micromol/kg of L-[3,3-^2^H_2_]cysteine (Cambridge Isotope Laboratories, Andover, MA) was started and continued for 7 hours. Arterial blood samples (for a total of 10 mL) were obtained at 3, 4, 5, 6 and 7 hours, to measure hematocrit and ^2^H_2_-enrichments of erythrocytes free-cysteine and glutathione [26]. Whole blood samples were immediately centrifuged and plasma and erythrocytes were separated. Erythrocytes were diluted with a known volume of cold distilled water. Plasma and erythrocyte solutions were frozen at -80°C for later analysis. Samples were processed as previously described [26]. In plasma samples we measured the concentrations of TBARS and in both plasma and erythrocyte the concentrations of glutathione and glutathione amino acid precursors (glycine, glutamine, glutamate, methionine, homocysteine, and cysteine in plasma; glycine, glutamine, glutamate, methionine, and cysteine in erythrocytes), as previously described [26].

### Analysis

Glutathione and cysteine isotopic enrichments were determined by gas chromatography-mass spectrometry (GCMS) (HP 5890, Agilent Technologies, Santa Clara, CA, USA) as referenced [26]. The N-methyl-N-tert-butyl-dimethysilyl-trifluoroacetamide derivatives were measured by electron impact ionization and selective ion monitoring at a nominal mass-to-charge ratio (m×z^-1^) of 363 and 365 for glutathione enrichment, and of 406 and 408 for cysteine enrichment. Total glutathione and precursor amino acid concentrations in erythrocytes, obtained from background samples, were determined using GCMS and the internal standard technique [23]. Plasma TBARS concentrations were measured using a commercially available kit (Oxitek, Zeptometrix Co, Buffalo, NY), following the manufacturer’s recommendations. Catalytic subunit expression of glutamate-cysteine ligase (GCL-C) in erythrocytes was measured by Western blot analysis, as described [26]. GCL-C protein concentrations were assessed as a ratio with glyceraldehyde-3-phosphate dehydrogenase protein.

### Calculations

Isotopic enrichments were expressed as tracer-tracee-ratio (TTR). Erythrocyte glutathione and amino acid concentrations were determined using standard calibration curves and normalized by erythrocyte volume obtained from hematocrit micromol/L(HCT)]. Glutathione fractional synthesis rate (FSR) (%/day) was calculated as follows:

$$FSR=100\times 24\times [E({}^{2}H_{2}glutathione)/E({}^{2}H_{2}cysteine)]$$

Where, E(^2^H_2_glutathione)/t is the slope (TTR/h) of the regression line describing the rise in erythrocyte ^2^H_2_-glutathione enrichment (TTR), as a function of time (hours), over the last 4 h of isotope infusion, and 24 and 100 convert FSR to percent/per day. Glutathione absolute synthesis rate was calculated by multiplying glutathione FSR by erythrocyte glutathione concentration [26]. During each study day, glycaemia levels, assessed every 2 h throughout the study, were plotted on a graph to geometrically calculate the area under the curve (AUC, mmol/L/h) from 6 values of blood glucose concentrations, as referenced [26]. Mean blood glucose concentrations were calculated during the last 6 hours of each study period. To evaluate relative variability of glucose, the coefficient of variability (%) was calculated for each patient as mean of standard deviations of glucose, divided by the mean, multiplied by 100 [27].

### Statistics

Data are expressed as mean±SEM. The effects of insulin-mediated glucose control on all variables were analyzed with ANOVA, using treatment (IIT and CIT) as within-subject factor and with protocol (1 and 2) as between-subject factor. Moreover, the effects on clinical data were also analyzed with a repeated measure analysis of variance (ANOVA), with time (day 1 and day 2) as within-subject factor. All comparisons were considered significant at p ≤ 0.05. Correlation between variables were analyzed by Spearman’s coefficient. Statistical analysis was carried out with SPSS software (version 21; SPSS Inc, Chicago, IL).

## Results

**Fig 2:**
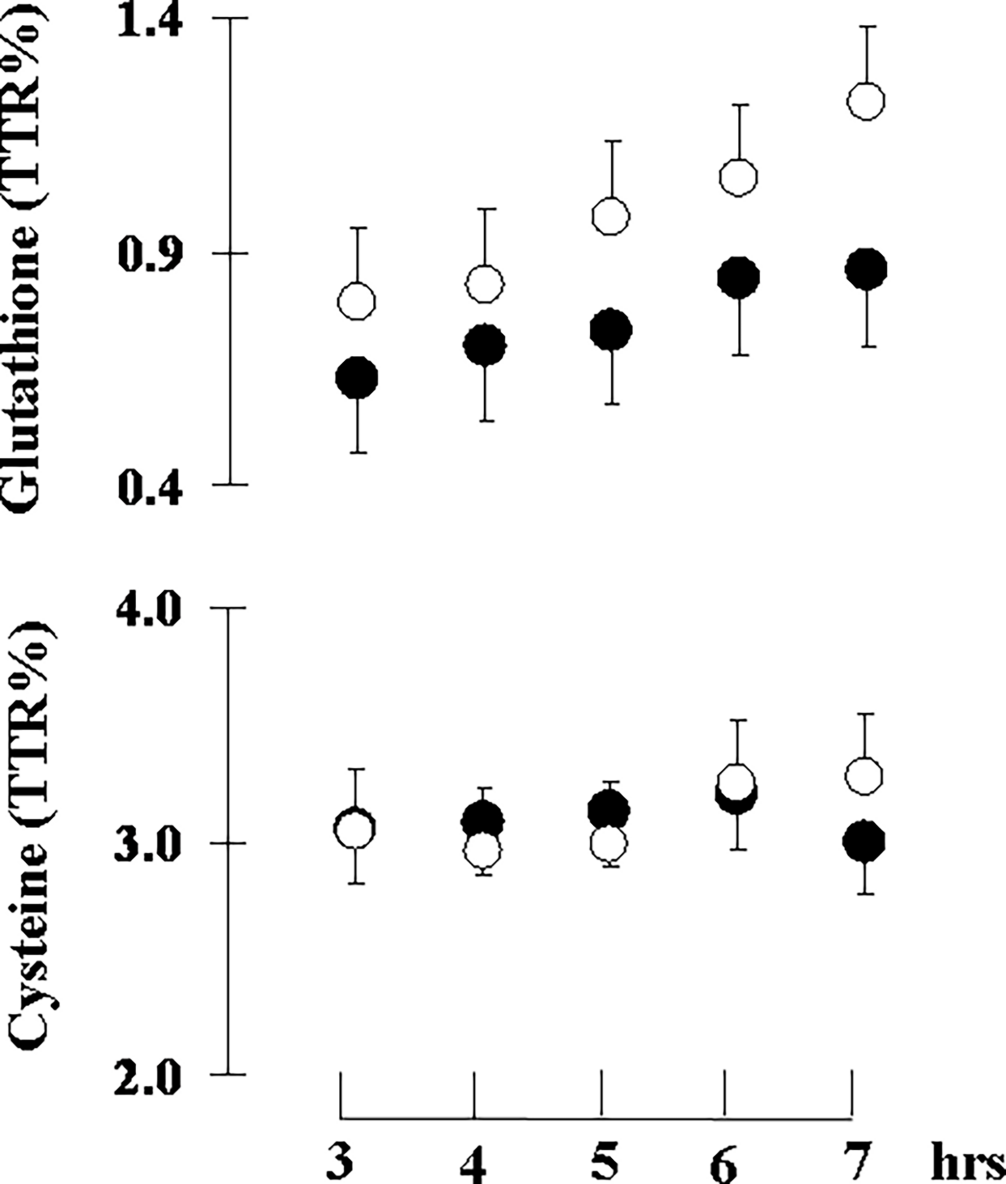
Glutathione kinetic. Mean±SEM precursor ^2^H_2_-cysteine and product ^2^H_2_-glutathione tracer-to-tracee ratios (TTRs) in erythrocytes during primed continuous infusion of ^2^H_2_-cysteine at the end of the control (○) and the intensive insulin treatment (●) periods.

**Fig 3:**
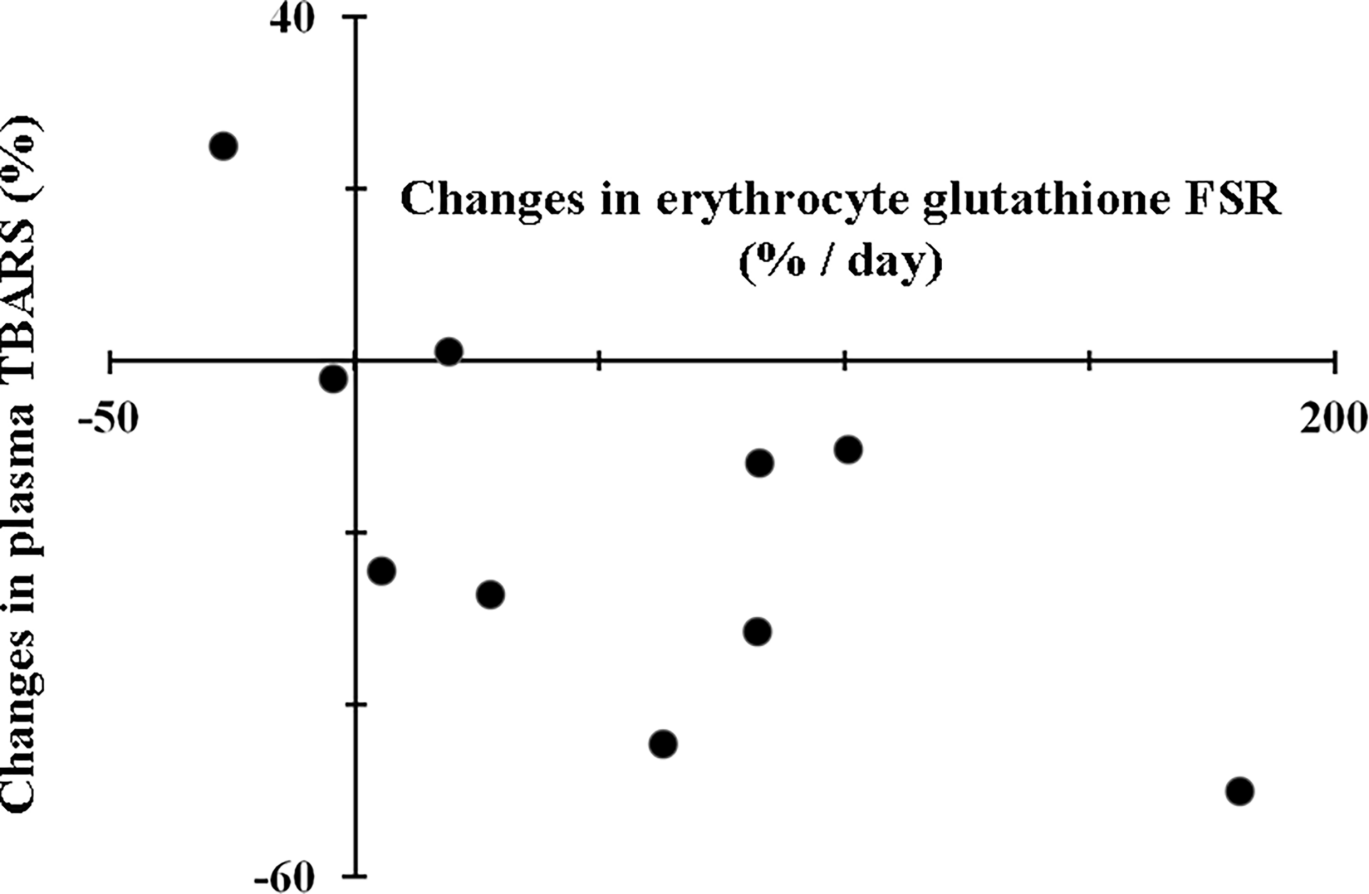
Relationship between insulin mediated changes in erythrocyte glutathione fractional synthesis rate (FSR) and plasma thiobarbituric acid reactive substances (TBARS). Spearman’s correlation coefficient, -0.69; n = 10; p = 0.03.

All patients completed the protocol and none of them were excluded for safety reasons. Hemodynamic and clinical chemistry indices showed no significant differences between IIT and CIT (Table 2). Nonetheless, there was a significant treatment×protocol interaction for hemoglobin levels. Hemoglobin significantly decreased by about 10% from study day 1 to study day 2 (12.0±0.6 versus 10.7±0.5 g; p = 0.035). The hematocrit followed the same trend of hemoglobin without achieving statistical significance. All patients required insulin infusion during both the IIT and the CIT (control) and none of them had hypoglycemia episodes (i.e., blood glucose < 3.9 mmol/L). IIT resulted in a tight glycemic control, while the conventional approach (CIT) led to moderate hyperglycemia (Table 3). Insulin administration was 70% lower during the control (CIT) than the IIT period (Table 3). There were no significant protocol effect or treatment×protocol interactions for insulin administration. Area under the curve of glucose concentrations (AUC_glycaemia_) was significantly lower with IIT than with CIT (Table 3). There were no significant treatment×protocol interactions for the AUC_glycaemia_. The coefficient of variability of glucose was no significantly different during IIT as compare to CIT. Precursor ^2^H_2_-cysteine enrichment was measured in erythrocytes and reached steady state in all groups and insulin treatments (IIT or CIT) by the end of the third hour of isotope infusion, whereas ^2^H_2_-glutathione enrichment in erythrocyte increased linearly with time (Fig 2). IIT significantly increased glutathione fractional (+218±81%) and absolute (+233±88%) synthesis rates in erythrocytes, being this result confirmed when normalized per hemoglobin content (Table 4). Moreover, IIT significantly decreased plasma TBARS concentrations (-18±7%) (Table 4). There were no significant treatment×protocol interactions for plasma TBARS and erythrocyte glutathione synthesis. IIT did not change significantly glutathione or glutathione precursor amino acid concentrations in erythrocytes (Table 5). Insulin-induced changes in erythrocyte glutathione fractional synthesis rates inversely correlated with changes in plasma TBARS concentrations at the end of each treatment (Fig 3).

**Table 2:** Clinical data during the experimental period.

	CIT	IIT	p	
			Treatment effect	Treatment × protocol interaction
SAP (mmHg)	143±5	136±6	0.95	0.81
DAP (mmHg)	67±2	65±3	0.46	0.33
Heart rate (beats×min^-1^)	86±3	83±4	0.37	0.77
Hematocrit (%)	33±1	32±2	0.78	0.15
Hemoglobin (g×ml^-1^)	11.6±0.4	11.2±0.7	0.18	0.002
Platelets (10^9^×L^-1^)	218±23	213±25	0.89	0.51
Leucocytes (10^9^×L^-1^)	11.5±1.8	10.1±1.6	0.40	0.07
Sodium (mmol×L^-1^)	138±1	138±1	0.72	0.55
Potassium (mmol×L)	3.71±0.07	3.92±0.10	0.49	0.11
Creatinine (mg×dL^-1^)	0.76±0.08	0.78±0.09	0.79	0.17
Urea (mg×dL^-1^)	31.0±2.1	33.2±3.6	0.49	0.53

Data are expressed as means±SEMs. SAP, systolic arterial pressure; DAP, diastolic arterial pressure. Data were analyzed with repeated measures ANOVA, with time as within subject variables (day 1 or day 2, after surgery) and sequence of treatments as between subject factors [Protocol 1 (n = 5), intensive insulin therapy (IIT) first, conventional insulin therapy (CIT) second; protocol 2 (n = 5), CIT first, IIT second)].

**Table 3:** Insulin infusion and glycemic control.

	CIT	IIT	p	
			Treatment effects	Treatment × protocol interaction
Insulin infusion (U×h^-1^)	1.2±0.5	4.0±1.0	0.03	0.15
Mean glucose concentration (mmol×L^-1^)	9.3±0.5	6.5±0.3	<0.001	0.49
AUC glucose (mmol×L^-1^×day^-1^)	223±12	154±7	0.002	0.40
Coefficient of variation of glucose (%)	9.7±1.4	10.5±1.1	0.43	0.56

IIT, Intensive insulin therapy; CIT, Conventional insulin therapy. Data are means±SEM of pooled values from protocols 1 and 2 obtained during the final 12 hours of CIT and IIT. AUC, area under the curve of blood glucose concentrations. Glucose variability throughout the experimental periods is expressed as mean (±SEM) of individual standard deviations (SD). Data were analyzed with repeated measures ANOVA, with treatment as within subjects variables (control and intensive insulin treatment) and sequence of treatments as between subjects factor [protocol 1 (n = 5), IIT first CIT second; protocol 2 (n = 5), IIT first, CIT second].

**Table 4:** Effects of intensive insulin treatment on systemic oxidative stress and glutathione synthesis.

	CIT	IIT	p	
			Treatment effects	Treatment × protocol interaction
Plasma TBARS (nmol×mL^-1^)	2.9±0.4	2.2±0.2	0.04	0.54
Erythrocyte glutathione FSR (%×day)	43±11	97±29	0.02	0.11
Erythrocyte glutathione ASR (mmol× L(HCT)^-1^×day^-1^)	1.18±0.29	2.70±0.51	0.01	0.12
Erythrocyte glutathione ASR (mmol×g(Hb)^-1^×day^-1^)	0.03±0.008	0.08±0.018	0.009	0.09
Erythrocyte GCL-C (fraction of GAPDH)	0.73±0.08	0.83±0.22	0.61	0.42

IIT, Intensive insulin therapy; CIT, Conventional insulin therapy; HCT, hematocrit; Hb, hemoglobin, TBARS, thiobarbituric acid reactive substances; FSR, fractional synthesis rate; ASR, absolute synthesis rate; GCL-C, glutamate cysteine ligase–catalytic subunit; GAPDH, glyceraldehyde-3-phosphate dehydrogenase. Data are means±SEM of pooled values from protocols 1 and 2 obtained at the end of CIT and IIT periods. Data were analyzed with repeated measures ANOVA, with treatment as within subjects variables (control and intensive insulin treatment) and sequence of treatments as between subjects factor [protocol 1 (n = 5), IIT first, CIT second; protocol 2 (n = 5), CIT first, IIT second].

**Table 5:** Effects of intensive insulin treatment on erythrocyte glutathione and precursor amino acid concentrations.

	CIT	IIT	P	
			Treatment effects	Treatment × protocol interaction
Glutathione [micromol×L(HCT)^-1^]	2783±183	3045±378	0.37	0.43
Glutathione [micromol×g(Hb)^-1^]	244.4±20.8	284.3±41.9	0.16	0.09
Cysteine [micromol×L(HCT)^-1^]	51±4	48±4	0.47	0.42
Glutamate [micromol×L(HCT)^-1^]	535±140	446±36	0.55	0.39
Glycine [micromol×L(HCT)^-1^]	448±23	469±20	0.11	0.81

IIT, Intensive insulin therapy; CIT, Conventional insulin therapy; HCT, hematocrit; Hb, hemoglobin. Data are means±SEMs of pooled values from protocols 1 and 2, obtained at the end of the control and the intensive insulin treatment periods. Data were analyzed with repeated measures ANOVA, with treatment as within subjects variables (control and the intensive insulin treatment) and sequence of treatments as between subjects factor [protocol 1 (n = 5), IIT first, CIT second; protocol 2 (n = 5), CIT first, IIT second]. There were no significant protocol effects for any of the parameters.

## Discussion

Glutathione synthesis rate is a key determinant of intracellular antioxidant capacity. We have shown in non-diabetic cancer patients that IIT, by preventing post-surgical stress hyperglycemia, was associated with increased glutathione synthesis and decreased systemic oxidative stress. Glutathione synthesis rates were measured in erythrocytes from the incorporation of labeled cysteine (precursor) into glutathione (product) [26]. Systemic oxidative stress was determined as plasma TBARS concentrations [27]. Insulin-mediated changes in glutathione synthesis rate correlated inversely with changes in plasma TBARS concentrations, showing the role of insulin or of changes in glycaemia levels in the regulation of oxidative stress.

All enrolled patients developed hyperglycemia and required insulin treatment, after surgery and intra-operative radiation therapy. We used a cross-over experimental design to compare the effects of conventional (CIT) or intensive (IIT) 24-h insulin therapy, given in randomized order, over 48-h after surgery [25]. IIT resulted in a tight glycemic control, while CIT led to moderate hyperglycemia. The sequence of post-surgical insulin treatments was randomized to prevent potential time-related metabolic effects. Since there were no differences between the two protocols, euglycemia results from IIT, either on day 1 or 2 after surgery, were pooled and compared with pooled hyperglycemia data from CIT), either on day 1 or 2 after surgery. Thereafter the statistical interactions between time after surgery and insulin treatment were assessed [25]. Despite the fact that we found no insulin/euglycemia effects on hemoglobin levels, there was a significant treatment × protocol interaction. In fact, there was a significant 10% decrease in hemoglobin levels from study day 1 to study day 2 due to fluid therapy. The hematocrit followed the same trend of hemoglobin without achieving statistical significance.

In our study, both IIT and CIT were characterized by absence of hypoglycemic episodes and by low-glycemic variability. It is well known that both glycemic excursions and hypoglycemia are major determinants of ROS production, redox system unbalance and glutathione depletion [28–31]. Moreover, mild hypoglycemia, as defined by blood glucose < 3.9 mmol/L, was associated with increased morbidity and mortality in ICU patients [32,33]. In our study, the ratios of glycemic variations, compared to the mean patient’s values, calculated as coefficient of variability, were about 10%, both in IIT and CIT. In a large cohort of 7049 mixed ICU patients the mean coefficient of variability of plasma glucose was about double than that observed in our study [33]. Glucose variability is an independent predictor of morbidity and mortality in ICU [8,34]. Continuous blood glucose–monitoring devices and computer-assisted closed loop systems for insulin delivery have been shown to prevent hypoglycemia and reduce glucose variability in ICU settings [17,18].

Previous evidence indicates that tissue glutathione synthesis capacity is decreased by surgical trauma [21]. The enzyme glutamate-cysteine ligase, which catalyzes the first and rate limiting reaction involved in glutathione biosynthesis, starting from cysteine and glutamate, is induced by reactive oxygen species, but down-regulated by stress hormones, as glucocorticoids and catechol amines [20]. In the present study, insulin-mediated tight glucose control, after surgery, doubled glutathione synthesis rates in erythrocytes, as compared to the condition of relative insulin deficiency with moderate hyperglycemia. In perfect agreement with our results, insulin infusion to control hyperglycemia in experimental sepsis, increased glutathione content in the liver by 78% [29]. Evidence indicates that glutathione metabolism, as assessed in the erythrocytes, may reflect the conditions of both whole body and liver glutathione system [20]. Expression levels of catalytic subunits of the glutamate-cysteine ligase enzyme did not change following CIT or IIT, suggesting a post-translational regulation of enzyme activity by insulin and/or changes in glucose levels. The differential effects of insulin and glucose on glutamate-cysteine ligase activity have been assessed in experimental models of insulin deficiency. Insulin can directly activate this enzyme and stimulate glutathione synthesis, independently from glucose concentration [24]. In addition to de novo synthesis, other mechanisms can affect intracellular glutathione pool and turnover. Detoxifying reactions involving generation of glutathione-S-conjugates by glutathione-S-transferases can decrease intracellular glutathione pools [20]. These pathways are activated by trauma and infections, as well as by insulin depletion and hyperglycemia, leading to accelerated glutathione disposal [20,30]. High levels of blood glucose are typically accompanied by increased production of free radicals, independently from insulin availability in patients with type 2 diabetes or stress hyperglycemia [20, 35]. In our study, therefore, either insulin-mediated improvement of anti-oxidant defenses or lower free radical production, secondary to euglycemia, could have contributed to decreasing plasma TBARS, following surgical stress. The observed inverse correlation between changes in glutathione synthesis rate and TBARS, suggests a cause-effect relationship between increased glutathione availability and improved redox balance.

We have used plasma TBARS concentrations as index of lipid peroxidation and systemic redox unbalance. Insulin-mediated tight glycemic control (IIT) significantly decreased TBARS concentrations by 15%, as compared to conventional insulin treatment (CIT) and moderate hyperglycemia. Both IIT and CIT may have contributed to decreasing systemic oxidative stress. In population-based studies, glucose levels directly correlated with TBARS in healthy individuals and in type 2 diabetics [34]. In physiological conditions, a diet with a high glycemic index was associated with greater oxidative stress, as measured by lipid peroxidation markers [36]. Tight metabolic control reduced TBARS in type 1 diabetic individuals [37]. Thus, our results are in perfect agreement with previous evidence suggesting a direct contribution of euglycemia in lowering lipid peroxidation markers.

## Conclusion

Stress hyperglycemia, independent from previous history of diabetes mellitus, is a frequent finding after major surgery, with a strong impact on patient’s outcome. We investigated the beneficial effects of IIT on oxidative stress in post-surgical patients. Our results underscore the potential of IIT in post-operative ICU patients to stimulate glutathione synthesis and decrease systemic oxidative stress, in the absence of hypoglycemic episodes and with low-glycemic variability. These findings have significant clinical implications since surgical trauma decreases the synthetic capacity and availability of glutathione [21] increasing morbidity and mortality [22].
